# Extracellular Vesicle Isolation Yields Increased by Low-Temperature Gaseous Plasma Treatment of Polypropylene Tubes

**DOI:** 10.3390/polym12102363

**Published:** 2020-10-15

**Authors:** Matic Resnik, Janez Kovač, Roman Štukelj, Veronika Kralj-Iglič, Petr Humpolíček, Ita Junkar

**Affiliations:** 1 Department of Surface Engineering, Jožef Stefan Institute, Jamova 39, SI-1000 Ljubljana, Slovenia; janez.kovac@ijs.si (J.K.); ita.junkar@ijs.si (I.J.); 2 Laboratory of Clinical Biophysics, Faculty of Health Sciences, University of Ljubljana, Zdravstvena pot 5, SI-1000 Ljubljana, Slovenia; roman.stukelj@zf.uni-lj.si (R.Š.); veronika.kralj-iglic@zf.uni-lj.si (V.K.-I.); 3 Centre of Polymer Systems, Polymer Centre, Tomas Bata University in Zlin, T.G. Masaryk Sq. 5555, 760 05 Zlin, Czech Republic; humpolicek@utb.cz

**Keywords:** atmospheric pressure plasma jets (APPJs), extracellular vesicles (EVs), nanostructures, polypropylene (PP)

## Abstract

Novel Extracellular Vesicles (EVs) based diagnostic techniques are promising non-invasive procedures for early stage disease detection which are gaining importance in the medical field. EVs are cell derived particles found in body liquids, especially blood, from which they are isolated for further analysis. However, techniques for their isolation are not fully standardized and require further improvement. Herein modification of polypropylene (PP) tubes by cold Atmospheric Pressure Plasma Jet (APPJ) is suggested to minimize the EVs to surface binding and thus increase EVs isolation yields. The influence of gaseous plasma treatment on surface morphology was studied by Atomic Force Microscopy (AFM), changes in surface wettability by measuring the Water Contact Angle (WCA), while surface chemical changes were analyzed by X-Ray Photoelectron Spectroscopy (XPS). Moreover, PP tubes from different manufacturers were compared. The final isolation yields of EVs were evaluated by flow cytometry. The results of this study suggest that gaseous plasma treatment is an intriguing technique to uniformly alter surface properties of PP tubes and improve EVs isolation yields up to 42%.

## 1. Introduction

Due to a large increase in the human population and demand for healthcare, novel fast, and effective techniques for diagnosis and treatment are needed [1]. Early stage disease diagnostic is of crucial importance for successful treatment and improved healing rates [2]. While traditional techniques are often invasive, target-specific, and can only be employed when the disease has already matured, novel Extracellular Vesicles (EVs) based techniques represent the opposite. EVs are small cell derived particles involved in cell-to-cell communication [3,4]. They carry the biological material from the cell of origin and are transported throughout the human body by all body fluids. Many studies suggest that EVs are promising biomarkers for diseases, such as metastasis of cancer, diabetes, coronary diseases, inflammatory diseases, infectious diseases, neurologic diseases, and others [2,5,6,7,8,9,10,11,12]. Furthermore, EVs can be collected rather un-invasively and give precise information about the infected cells from which they originate. It is important to be aware that EVs are not abundant and are hard to isolate due to their small size (100–1500 nm) and sensitivity [13]. Isolation of EVs currently represents a major drawback since EVs are likely to interact with surfaces of medical tools. The interaction leads to depletion of the EVs in liquids merely by irreversible binding onto the surface of the tubes or other medical tools used for collecting, sampling, storage, transport, and isolation of EVs. Especially critical are the surfaces of 1.5-mL polypropylene (PP) tubes, also known as Eppendorf tubes (hereon referred only as tubes) where blood or other body fluids are centrifugally processed and stored. The above mentioned tubes are standardized transparent conical snap-cap tubes manufactured by different suppliers and used in laboratories worldwide. Their surfaces are hydrophobic which is favorable for some applications but not for ultra-centrifugal isolation of EVs [14].

The hypothesis of the present work is, that the prevention of excessive binding of EVs to the tube wall can be achieved by appropriate gaseous plasma treatment of the inner tube surface. Decreased binding of EVs to the tube wall would therefore enable higher isolation yields of EVs, which should improve the diagnostics of these precious biomarkers. Additionally, increased wettability of tube surfaces should reduce shear stress and prevent the disintegration of EVs during the isolation process. The change in morphology after gaseous plasma treatment of tubes might also create a more profound surface film, again, preventing the EVs from coming into direct contact with tube surfaces.

**Figure 1:**
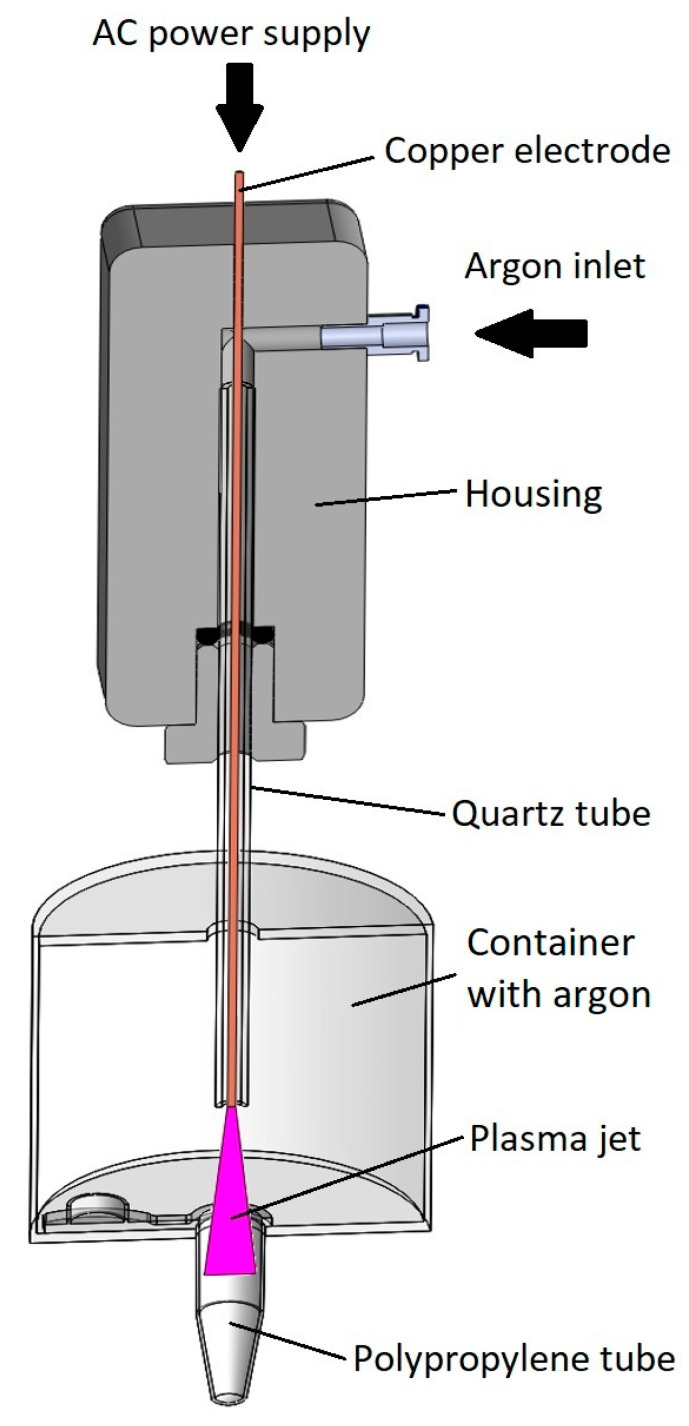
Schematics of gaseous plasma treatment of tube inside a container in cross-section view.

Gaseous plasma treatment of polymers has been a valuable tool for changing surface characteristics, such as nanostructure, chemical composition, and surface energy, for decades [15,16,17]. The invention of cold Atmospheric Pressure Plasma Jet (APPJ) sources has revolutionized various fields including medicine [18,19]. Direct and indirect gaseous plasma treatment appear in medical applications ranging from chronic wound healing, cancer therapy, improving implant biocompatibility, decontamination of surfaces, and more [20,21,22,23,24,25]. In the case of direct gaseous plasma treatment, living cells are in contact with gaseous plasma species while in the case of indirect the contact is between gaseous plasma treated surfaces or liquids and cells. The latter refers to modification of internal surfaces of tubes in such a manner that hydrophilicity is improved and morphology changes to a more favorable, evenly nanostructured one. The treatment of internal surfaces of a long tube with gaseous plasma jet is a challenge by itself. Furthermore, curved surfaces present a problem for most analytic techniques. Hence most research groups decide to use foil made of what is declared to be the same polymer, due to more convenient surface analysis. In our case vast differences already appeared between tubes from different manufacturers, therefor foil was not an option. It also has to be considered that the dynamic of APPJ treatment cannot be simulated on a flat surface due to complex mechanisms of gaseous plasma activated species being transported in a unique manner inside the tube. These species and their interaction with the surface are of crucial importance when mechanisms of surface functionalization and etching are studied. The main requirement for successful tube modification is that all surfaces are evenly treated by gaseous plasma. This was achieved by below listed settings:

- The vertical position of the gaseous plasma jet ensures that the ground is coaxially positioned with the axis of symmetry. In the case of horizontal setup lower part of the tube was more effectively treated by gaseous plasma compared to the upper part due to the jet being attracted to the ground (Figure 1).
- Gaseous plasma treatment of the tube was conducted inside an enclosed container to ensure argon atmosphere with less quenching, as in this setting plasma jet is wider and less focused, streamers are more branched. Gas flow is initiated before the plasma jet is turned on. Argon is heavier than air and displaces some of the air from the bottom of the container.
- Treatment is not stationary, the plasma jet is moving in and out of the tube during treatment. The movement is enabled by a linear stage to ensure equal speeds and acceleration/deceleration and consequently even and repeatable gaseous plasma treatment.
- Gas flow is optimized in a manner that the plasma jet is stable.

## 2. Materials and Methods

### 2.1. Preparation of Samples

APPJ with a single electrode was used for the treatment of tubes. It was powered by a commercial 25 kHz high voltage Alternating Current (AC) electrical source. Plasma jet output parameters were set to 2.5 kV and 3 mA. The electrode was a 0.1 mm diameter copper wire inside a 60-mm long quartz tube with an inner diameter of 1.2 mm. Argon was used as a working gas and was leaked through the previously mentioned quartz tube at 2 slm with a flow controller MV-304 (Bronkhorst, Ruurlo, Holland). The plasma jet was mounted on top of the linear stage while the tube was coaxially fixed inside an enclosed container shown in Figure 1. The container was filled with argon prior to treatment, to ensure a controlled atmosphere. During the treatment, the plasma jet was traveling in and out of the tube five times, complete treatment time being 15 s.

Tubes from three different manufacturers were used in this experiment. They were labeled as Sample 1, Sample 2, and Sample 3 and were obtained from AHN Biotechnologie GmbH (Nordhausen, Germany), Ratiolab GmbH (Dreieich, Germany), and Eppendorf GmbH (Hamburg, Germany), respectively. All of them were standard PP 1.5 mL conical transparent tubes with a snap-cap. These particular tube manufacturers were chosen on the basis of preliminary experiments, due to differences in chemical composition, morphology, and water contact angle of their tubes, to observe how these features affect the EV isolation. Gaseous plasma treatment took place approximately one hour before EV isolation to avoid aging effects.

### 2.2. X-ray Photoelectron Spectroscopy

Inner surface functionalization of gaseous plasma treated tubes was determined by X-Ray Photoelectron Spectroscopy (Physical Electronics, Munich, Germany). Freshly gaseous plasma treated tubes were cut in smaller pieces and put inside XPS within an hour after treatment. The samples were excited with monochromatic Al Kα_1,2_ radiation at 1486.6 eV over an area with a diameter of 400 µm and approximately 5 µm in depth. Photoelectrons were detected by a hemispherically shaped analyzer, which was positioned at a 45-degree angle with respect to the normal of the sample surface. High-resolution spectra were measured at a pass energy of 23.5 eV using an energy step of 0.1 eV, whereas survey spectra were measured at a pass energy of 187 eV using an energy step of 0.4 eV. Surface neutralization during XPS measurements was achieved by the additional electron gun. T main C1s peak of the carbon atoms was used as the reference for all spectra and was assigned a value of 284.8 eV. All measured spectra were processed and analyzed using MultiPak v8.1c software (Ulvac-Phi Inc., Kanagawa, Japan, 2006) provided by Physical Electronics.

### 2.3. Atomic Force Microscopy

Changes in surface morphology of tubes were analyzed by Atomic Force Microscope Solver PRO (NT-MDT, Moscow, Russia) in tapping mode in air. Samples were cut in small pieces and the inner surface was scanned by a standard Si cantilever with a force constant of 22 N m^−1^ and at a resonance frequency of 325 kHz. The cantilevers’ tip radius was 10 nm, the tip length was 95 µm and the scan rate was set at 1.3 Hz. Every measurement was repeated at least 5 times. Average surface roughness (Sa) was measured from representative images on 10 × 10, 5 × 5, 2 × 2, and 1 × 1 µm^2^ areas with the Nova AFM software (NT-MDT, Moscow, Russia). Only 1 × 1 area results are shown in Table 3. Prior to this study, treatment parameters were optimized and AFM measurements were performed on different depths of gaseous plasma treated tube to confirm that treatment effects are even.

### 2.4. Differential Scanning Calorimetry

Differential scanning calorimetry (DSC) was used to investigate the melting and crystallization behavior of PP tubes from different manufacturers. The samples were investigated using DSC821^e^ (Mettler Toledo, Columbus, OH, USA) at heating and cooling rates of 10 °C min^−1^ between 20 °C and 180 °C. The samples were purged with nitrogen at a flow rate of 50 mL/min^−1^. Melting enthalpy was obtained from the melting peak area, while the tube crystalline fraction was calculated by dividing the melting enthalpy by fusion energy of 100% crystalline PP (H = 207.1 J g^−1^) [26]. All data was processed with a STAR SW 14.0 software (Mettler Toledo, Columbus, OH, USA).

### 2.5. Water Contact Angle

Water Contact Angles (WCAs) of inner surfaces of tubes were measured by See System E (Advex Instruments, Brno, Czech Republic) equipped with a CCD camera connected to a PC. For this purpose, gaseous plasma treated tubes were cut in half along the symmetry axis. Afterward, a 2 μL droplet of demineralized water was placed on the inner surface and a high resolution image was taken. WCAs were than determined by the See System 7.0 software (Advex Instruments, Brno, Czech Republic) with a three-point fitting method. At least 6 measurements were performed for each untreated and treated tube. WCAs on treated samples were measured within one hour after gaseous plasma treatment as that is also the time in which they are used for EV isolation.

### 2.6. Blood Sampling

The study was given ethical permission from the National Medical Ethics Committee (number 82/07/14) and it conformed to the ethical principles given in the Declaration of Helsinki. Blood was obtained from 10 healthy adults (3 females, average age 32 ± 4 years and 7 males, average age 30 ± 5 years) with their written consent. Sampling was performed after at least 12 h of fasting. Each subject donated blood by venipuncture into two 2.7 mL vacutubes containing 270 μL 0.109 mol L^−1^ trisodium citrate (Becton Dickinson, Franklin Lakes, NJ, USA) and was gently mixed by turning the tube upside down multiple times. Prior to sampling, tubes were prewarmed to 37 °C and samples were kept in thermoblocks to minimize the activation of platelets caused by thermal stress [27,28]. Centrifugation of the samples started within 15 min after the acquisition of the first sample.

### 2.7. Isolation of EVs

In order to separate the cells from blood plasma, samples were centrifuged at 1550× *g* for 20 min in a Centric 400/R centrifuge (Tehtnica, Železniki, Slovenia). The centrifuge was prewarmed to 37 °C. Blood plasma was removed slowly from both vacutubes and placed in a 4 mL glass tube, where it was homogenized on vortex at 1200 rpm for 5 s. One point five milliliter of blood plasma sample was divided into 6 parts, 250 μL each, for further centrifugation. Gaseous plasma treated and untreated PP tubes made by three different manufacturers (mentioned before) were used. The samples were then centrifuged at 17,570× *g* for 5 min in a Centric 200/R centrifuge (Tehtnica, Železniki, Slovenia). The supernatant (210 μL) was discarded while pelleted EVs (40 μL) were gently washed by 210 μL of citrate and Phosphate Buffered Saline (PBS, pH 7.4 at room temperature). Samples were centrifuged again at 17,570× *g* for 5 min. The supernatant (210 μL) was discarded while pelleted EVs (40 μL) were resuspended by the addition of 60 μL PBS.

### 2.8. Flow Cytometry

MACSQuant Analyzer (Miltenyi Biotec GmbH, Bergisch-Gladbach, Germany) flow cytometer with 405 nm, 488 nm, and 640 nm air cooled lasers was used for determination of EVs concentration in isolates. For data acquisition and result analysis, the MACSQuantifyTM software (Miltenyi Biotec GmbH, Bergisch-Gladbach, Germany) version 2.4 was used. Twenty-five microliters of the sample was measured for each instance. The presence of residual cells and EVs was determined by forward and side scatter parameters. The target area was a region of events corresponding to EVs, also named EVs density plot. For measurement to be statistically relevant, it must contain more than 90% of events in EVs density plot. All of the measurements presented herein, contain over 90% of events in EVs density plot.

## 3. Results

### 3.1. X-ray Photoelectron Spectroscopy

The surface chemistry of the tubes’ top surface layer was analyzed by X-ray Photoelectron Spectroscopy (XPS). The chemical composition of the tube surface is given together with the O/C ratio in Table 1. As can be seen from Table 1, Sample 3 has almost no oxygen present on the surface prior to gaseous plasma treatment, which would be expected for PP, as no oxygen is present in its chain. However, other PP samples have about 8 to 10 at% of oxygen on the surface. The moisture and oxygen from the atmosphere are removed by a high vacuum inside the XPS; thus, oxygen content on the surface is relevant and could be observed due to various fillers used in polymer manufacturing. Results of XPS analysis done on different samples indicate that PP samples differ in surface composition, which does not only influence the biological interactions, but also the interaction of the surface with gaseous plasma species. Higher oxygen content on the surface of all tubes was observed after gaseous plasma treatment; however, it seems that the highest concentration of oxygen on gaseous plasma treated surfaces was about 9 to 12 at%. These results are in good agreement with most of the topic-related papers reporting concentrations around 9 to 15.1 at% of oxygen, with two exceptions [29,30,31,32]. Kwon et al. report the highest concentration at 42.9 at% of oxygen on the PP film after argon plasma treatment at atmospheric pressure [33]. This is probably the result of PP film preparation prior to gaseous plasma treatment and the use of much higher gaseous plasma treatment power and longer treatment time. Second is Kostov et al., who reported 27 at% of oxygen on PP surface after atmospheric plasma treatment prior to rinsing with water. The concentration of oxygen drops to 12.5 at% after rinsing [31]. It is suggested that rinsing removes Low Molecular Weight Oxidized Material (LMWOM) which are loosely bonded and are mainly responsible for hydrophobic recovery.

Nevertheless, after gaseous plasma treatment, all samples exhibit a similar increase in the amount of oxygen on their surface, regardless of the initially different oxygen content. The oxygen functionalization is achieved by the surrounding air (present inside the enclosed container and mixed with argon) which is excited and made reactive by excited and ionized argon species from the plasma jet [34]. The competition between functionalization and etching is taking place during plasma treatment and saturation with oxygen functional groups seems to be reached at this treatment conditions on all surfaces. However, it should be pointed out that the influence of plasma etching differs between samples, as shown hereinafter.

**Figure 2:**
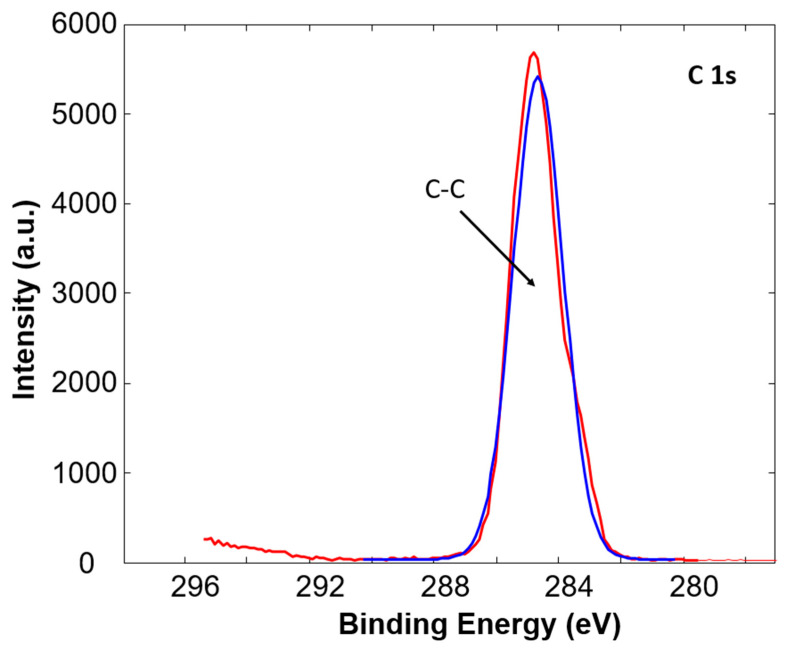
High resolution XPS spectra of untreated Sample 3.

**Figure 3:**
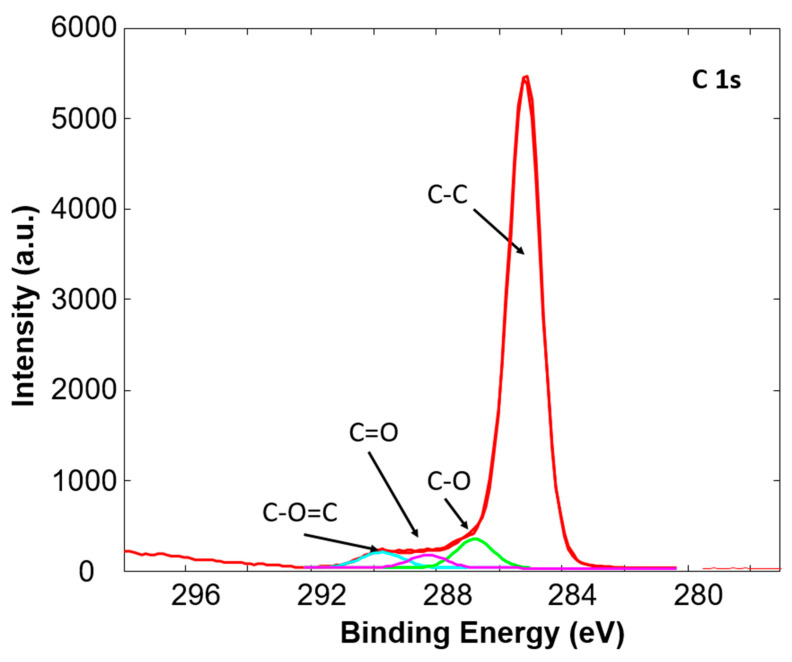
High resolution XPS spectra of plasma treated Sample 3.

In addition, the high resolution C1s peaks were recorded for a detailed analysis of all samples. The example of a C1s peak for untreated and plasma treated Sample 3 is presented in Figure 2 and Figure 3, respectively. The C1s peak of the untreated Sample 3 consists of one peak at a binding energy of 284.8 eV corresponding to C-C and C-H bonds in the polypropylene polymer chain. After oxygen plasma treatment the change in high resolution C1s peak is evident, as shown in Figure 3. The C1s peak is broader and the shoulder at a binding energy of 288.9 eV is observed. After deconvolution of the peak it can be observed that the peak consists of three peaks; a peak at a binding energy of 284.8 eV corresponding to C-C and C-H bonds, a peak at a binding energy of 286.6 eV corresponding to C-O bond, and a peak at a binding energy of 288.9 eV corresponding to O=C-O bond [35]. The increase in the latter two peaks after plasma treatment is due to the incorporation of oxygen into the surface of the samples. Similar was observed also for other PP tubes. The concentration of functional groups on the untreated and plasma treated samples is presented in Table 2. It can be observed that an increase in C-O, as well as O-C-O bonds, occur in all samples, while the most prominent increase is observed in the case of Sample 3, which initially had almost no oxygen on the surface.

### 3.2. Atomic Force Microscopy

**Figure 4:**
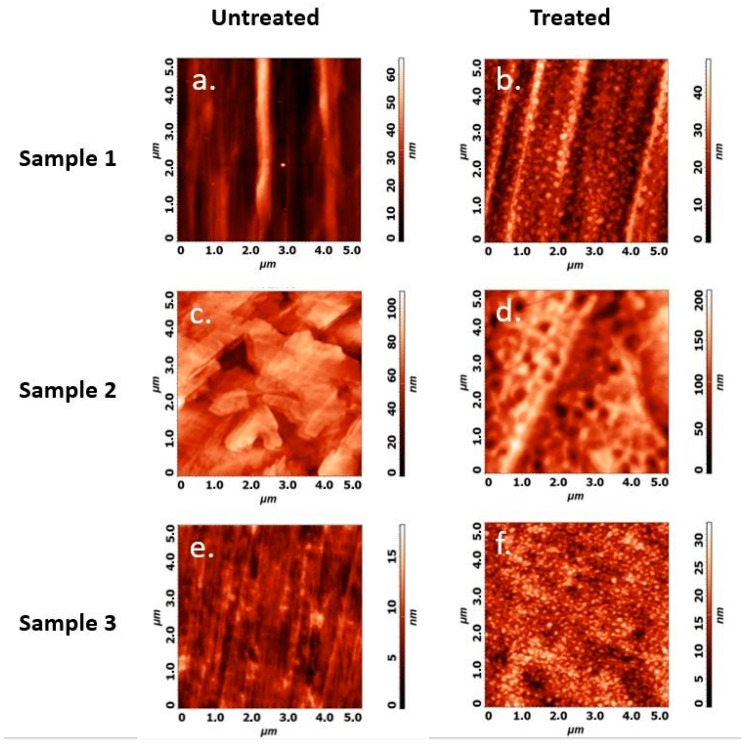
Two-dimensional Atomic Force Microscopy (AFM) images showing gaseous plasma nanostructured surfaces measured on 5 × 5 µm areas. (**a**) untreated sample 1; (**b**) treated sample 1; (**c**) untreated sample 2; (**d**) treated sample 2. (**e**) untreated sample 3; (**f**) treated sample 3.

Morphology of inner tube surfaces was investigated by Atomic Force Microscopy (AFM) and major differences were observed as seen in Figure 4. Samples 1 and 3 have similar morphology with razes from the manufacturing process all aligned in one direction. After gaseous plasma treatment razes are still present but due to uneven etching of the polymer matrix, small grain-like structures and craters appear. This is mainly correlated with differences in the etching of amorphous and crystalline parts, as it was already discovered that amorphous parts of polymer are more easily etched compared to its crystalline counterparts [36,37]. The initial morphology of Sample 2 (Figure 4c) indicates that this sample has different morphology compared to the other two samples. It has a sheet-like structure, which was not observed for the other two samples and is supposed to be the result of the manufacturing process. However, the average surface roughness of Sample 2 is similar to Sample 1. The influence of gaseous plasma etching of Sample 2 (Figure 4d) is also different, as grain-like structures were not formed on the surface and only craters with about 350 nm in diameter and 70 nm of depth were formed. In the case of gaseous plasma treated Sample 1 and Sample 3 (Figure 4b,f), the grain-like structures are approximately 250 and 120 nm in diameter and about 10 and 8 nm in height, respectively. Grain-like structures are more commonly reported with grains growing with increased gaseous plasma treatment time until a certain threshold [31,33,38]. After this threshold, the grains are etched away and the process of grain growth is periodically repeated [29]. Mean surface roughness results shown in Table 3 suggest that roughness has increased after gaseous plasma treatment as expected. It is interesting to observe that untreated Sample 3 has a really flat surface, as initial roughness is only about 0.74 nm, while higher roughness of about 2.4 nm is measured on Sample 1 and Sample 2. This could be mainly attributed to the manufacturing process. Similar initial morphology is observed when comparing Sample 1 and Sample 3. The main difference is in a wavy morphology of Sample 1, while a flat surface is observed for Sample 3.

### 3.3. Differential Scanning Calorimetry

Results from DSC analysis indicate that PP tubes from different manufacturers have different degrees of crystallinity, as seen in Table 4. Sample 2 has the lowest degree of crystallinity, about 42%, followed by Sample 1 with about 54% and Sample 3 with 58%. This can be correlated with plasma etching of PP tubes, as it seems that Sample 2 is etched differently compared to Sample 1 and Sample 3. According to AFM analysis, the surface of Sample 2 does not have a small grain-like structure after plasma treatment, as observed on Sample 1 and Sample 3. It has already been shown that plasma etching of polymers is influenced by the degree of crystalline fractions, as the amorphous parts are preferentially etched compared to its crystalline counterparts [24]. Thus, the degree of crystallinity in polymers can significantly influence the final surface nanotopography and further surface interaction with the biological environment (EVs, cells, etc.).

### 3.4. Water Contact Angle

**Figure 5:**
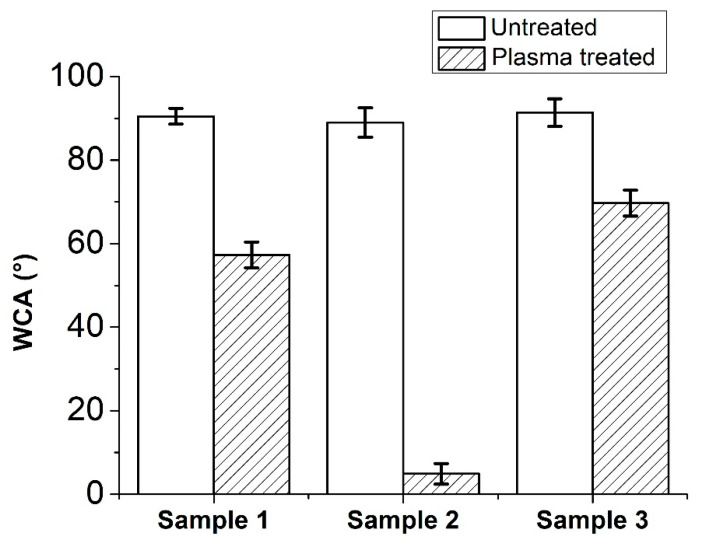
Water contact angle comparison between three samples in untreated and gaseous plasma treated state.

WCA measurements were used as the first indicator of gaseous plasma treatment effects. All samples exhibit similar wettability of around 90 degrees in an untreated state (Figure 5). As it is expected, WCA decreases for all samples after argon plasma treatment. It is noteworthy to mention how Sample 2 became almost super hydrophilic after a relatively short gaseous plasma treatment. This is definitely not what one might expect from PP, at least not according to the literature. The lowest WCA achieved by atmospheric pressure argon plasma on PP was reported to be 30 degrees by Akishev et al. [39]. WCA results for Sample 1 and 3 are in line with those reported in the literature [30,31].

### 3.5. Flow Cytometry

In order to compare results from different subjects, the percentage of EV isolation improvement was calculated. EVs from untreated tubes stand for 100% and improvement was calculated using Equation (1):

(1) $$Improvement\;\%=\left(\frac{EVs\;plasma\;treated\;tube}{EVs\;untreated\;tube}-1\right)\times 100\%.$$

The average results of all subjects together are presented in Table 5. As can be seen, Sample 1 has both the highest improvement rate and lowest standard deviation. Due to a small population of subjects, the standard deviation is high for all samples, but trends are still clearly visible. Further on, an improvement in isolation can be seen from EVs density plots, where untreated tubes averaged at 92% ± 3%, while gaseous plasma treated tubes manifest an improvement at 96% ± 3%. In addition, the size distribution of EVs suggests higher amounts of larger EVs in isolates from treated tubes and more EV debris from untreated tubes. This implies that indeed disintegration of EVs might have been prevented by gaseous plasma treatment.

Based on the results presented in this section, Sample 1 is superior to other samples due to its unique morphology and roughness. It has considerably higher roughness than the other two samples and an even morphology, which could help improve the formation of surface film and prevent the binding and disintegration of EVs during the isolation. It is believed that increased wettability also contributes to the improvement of surface film formation but not as much as morphology and roughness. The most hydrophilic sample, gaseous plasma treated Sample 2 is not as efficient when it comes to the isolation of EVs, hence increasing wettability must have its limits.

## 4. Conclusions

Polypropylene tubes made by different manufacturers were treated by atmospheric pressure plasma jet with argon as the feed gas. After a short treatment time of 15 s, surface morphology, chemistry, and wettability changes were observed.

Surface roughness increased as nano-sized grains and craters appeared on the surface due to gaseous plasma etching. Furthermore, the amount of oxygen on the surface is increased after gaseous plasma treatment, and all samples seem to reach their oxygen saturation level at about 9 to 12 at%. Sample 3 exhibits an increase of 10.9 at% of oxygen on the surface after gaseous plasma treatment, mainly due to a very low amount of oxygen on the untreated PP surface. Wettability increases dramatically in one case reaching almost super-hydrophilic properties.

Results from flow cytometry confirmed that the isolation yield of EVs is improved by 42%, 26%, and 17%, for treated Sample 1, Sample 3, and Sample 2, respectively. The size distribution of EVs is also improved for all treated tubes. The most important surface characteristics, responsible for improving EVs isolation yields are morphology and roughness. Increased wettability is also desired, but only up to a reasonable threshold. Further work is required to determine the exact role of other surface characteristics in improving EVs isolation yields by gaseous plasma treatment.

## 5. Patents

A patent covering the work presented herein was granted under the number WO 2016030358 A1.

## Figures and Tables

**Table 1:** All chemical elements detected by X-Ray Photoelectron Spectroscopy (XPS) with the corresponding O/C ratio.

		C1s (at%)	O1s (at%)	O/C
**Sample 1**	**Untreated**	91.5	8.5	0.09
	**Treated**	84.8	15.2	0.18
**Sample 2**	**Untreated**	90.2	9.8	0.11
	**Treated**	81.0	19.0	0.24
**Sample 3**	**Untreated**	99.4	0.6	0.01
	**Treated**	85.1	14.9	0.18

**Table 2:** Deconvolution of high resolution carbon (C1s) spectra.

		C-C (%)	C-O (%)	C=O (%)	O=C-O (%)
**Sample 1**	**Untreated**	96.3	2.4	0	1.2
	**Treated**	82.1	10.2	3.7	4.0
**Sample 2**	**Untreated**	90.4	7.4	0	2.2
	**Treated**	75.0	16.5	3.2	5.3
**Sample 3**	**Untreated**	100	0	0	0
	**Treated**	81.8	9.1	4.8	4.3

**Table 3:** Mean surface roughness Sa measured on 1 × 1 µm area by AFM.

	Untreated (nm)	Treated (nm)
**Sample 1**	2.48 ± 0.51	3.90 ± 1.32
**Sample 2**	2.40 ± 0.34	2.64 ± 0.52
**Sample 3**	0.74 ± 0.21	2.58 ± 0.05

**Table 4:** Differential scanning calorimetry (DSC) results, measured melting and crystallization temperatures, and calculated crystallinity.

	Tm (°C)	Tc (°C)	Crystallinity (%)
**Sample 1**	161.3	119.1	53.6
**Sample 2**	149.3	120.6	41.8
**Sample 3**	166.0	133.4	58.0

**Table 5:** Calculated improvement of Extracellular Vesicle (EV) isolation yields, combined.

	Improvement (%)
**Sample 1**	42 ± 23
**Sample 2**	17 ± 31
**Sample 3**	26 ± 38
